# The Physiology and Pathology of the Mouth

**Published:** 1860-01

**Authors:** 


### THE PHYSIOLOGY AND PATHOLOGY OF THE MOUTH.

Those whose professional labors in good part are devoted to counteracting and repairing derangements and diseases of the teeth, do not require any arguments to convince them of the importance of a clear and distinct understanding of the physiology and pathology of the buccal cavity; for the actions in health and in disease, of the other structures of the mouth, and of the entire alimentary canal even, have an important influence not only on the natural teeth, but also upon the materials used by dentists in repairing the ravages of disease.

The mouth, in common with the entire extent of the alimentary canal, is supplied with

Epithelium,
Blood-vessels and Blood,
Nerves,

Muscles,
Mucus,
Water,
Media Solution.

It also has,

Glands, and
Saliva, in addition to the more solid portions, which, however, have far more to do with the *Anatomy* than with the Physiology of the mouth.

The *Epithelium* of the mouth is in many regards like the *skin* which covers the body externally, but it is quite thin, delicate, and translucent, lying upon, yet scarcely concealing the papilæ. It is made up of minute *cells* whose sides are closed to each other and thus form *scales*, which are tipped down and lie upon each other very much like tiles upon a roof, in layers, and not in a mass. Each of these scales retains its characteristics as an individual scale, with an interval between it and its neighbor, and yet insufficiently intimate that the mouth may be subjected to a considerable amount of hard usage and yet receive no apparent injury, and the scales themselves not materially changed by ordinary mechanical or chemical impressions.

This entrance to the *prima vie* appears to be *paved*, so as to allow hot or cold food and drinks, acids and alkalies, fluids and solids of all kinds to pass on these scales over the papillæ, without making any apparent impression upon those delicate structures, while the interspaces between the pavement scales allow a free passage of the fluids in the mouth to be absorbed, or the fluids within the tissues to pass into the mouth. These epithetical scales are greatly modified in character and appearance by the nature of the fluid by which they are acted upon; those changes being the result of the action of the fluids upon the scales, and not of the epithelium upon the fluid.

On the tongue, the power of the mucous membrane to *form*

epithelium is manifested in a marked degree. Usually large numbers of epithetical scales can be scraped off with the blade of a knife. And as this is very much more marked upon the tongue than upon parts of the mucous membrane lower down in the intestinal tube, a slight change in the stomach becomes quite apparent in the changed appearance of what is called the *coating* of the tongue.

If the epithelium is formed too rapidly, and is so tenacious as not to be readily wiped off with food, and especially if the individual does not take into his mouth any solid food to wipe off the epithelium, it must coat the tongue. If the end of the cell or scale remains quite adherent to the skin, while there is considerable moisture in the mouth, so that the scales do not lie down, but stand on end, like the pile on velvet, the tongue is said to be "furred." If the scales are less adherent, and fall down between the papillæ, become soft and swollen, the tongue will be even, smooth on its surface, and "creamy." If the epithelium is rapidly thrown off, or not readily formed, and especially if the parts beneath are inflamed, and the tongue becomes dry, it becomes red and "raw." If the blood does not circulate through the tongue readily from general debility, a loss of blood, or other cause, the tongue spreads out, becomes flabby, receives the impressions of the teeth, and has a "pasty" appearance.

We are not to suppose that the mouth alone is affected when these changes appear, but to reflect that the mouth is a part of the alimentary canal and in intimate relations with other parts of it and particularly with that portion of it above the pyloric orifice of the stomach, and that changes in other parts of the canal, quickly make themselves manifest by changes in the appearance of the mouth, and by observing the condition of the mucous membrane of the mouth we can often judge quite accurately of its condition lower down.

The *Blood-vessels* of the mucous membrane are almost infinite in multitude, and are situated immediately beneath the thin translucent epithelium, and are filled with blood, even the capillaries in this part of the system being of sufficient calibre to admit the free transmission of the red corpuscles of the blood. The blood, from the number of the red corpuscles in it is quite red, and the blood-vessels of the mucous membrane of the mouth are so very numerous that the amount of fluid within the vessels equals the amount of solids in the walls of the vessels and in the membrane, both combined. Hence the mucous membrane, including the blood, may be said to be half fluid.

But this fluid in the mucous membrane of the mouth, is by no means stagnant, as the waters of a marsh or bog, but circulates with a ceaseless activity which has much to do with the *absorption* of fluids from the mouth, for endosmotic absorption is greatly influenced both by the amount of surface of the enclosed fluid exposed to the endosmotic action, and the rapidity of the circulation of that fluid.

It is always well to bear in mind the conditions of governing endosmosis, and it is particularly important in connection with the physiology of the mouth in which endosmosis bears so important a part, and therefore the presentation of those *conditions*, in this connection, although somewhat digressive, can not be considered entirely out of place. In regard to the endosmotic circulation of fluids, through membranes, it has been observed:—

1. When a membrane is interposed between two fluids of different densities, the fluids will pass through the membrane in both directions, but the fluid will pass from the rarer fluid to the denser with far greater rapidity than from the denser to the rarer.

2. If the dense fluid is in motion, the activity of the current to it is increased in proportion to the activity of the motion in the denser fluid.

3. If the dense fluid is alkaline and the rare fluid is acid, the current through the membrane will be accelerated.

4. If the dense fluid is warmer than the rare one, the increase of temperature will also aid in increasing the activity of the flow into the warmer fluid.

Not only is the epethilial membrane filled with large blood vessels, but as is admirably shown in the plates of Harsall's Microscopical Anatomy, and by Ford and Bowman, reproduced by Kolliker and Salter, and others, but the papillæ are supplied with loops of vessels that make up a large proportion of their entire bulk, thus increasing the amount of surface of membrane exposed to the ormotic action.

The conditions that favor the passage of fluids from the mouth through the epithelium and the walls of the blood vessels into the blood, are: —

The blood is more dense than the fluid within the mouth,

The blood is in active motion,

The blood within the vessels is alkaline, while the fluid in the mouth is usually acid,

The blood is usually more warm than the fluids in the mouth.

As liquid is being constantly poured into the blood-vessels, the blood would become less dense, and absorption impeded, but that there is an erroneous evaporation of water constantly taking place from the lungs and the skin, and no inconsiderable drainage through the kidneys. The kidneys, the skin, and the lungs are thus constantly engaged in removing water from the blood as fast as it is supplied through the mucous membrane.

There are other circumstances that have an influence on this ormotic or membranous circulation which are worthy of note. The activity of the motion of the blood is greatly modified by the condition of the nervous system. The sudden suffusion of the cheeks with blushes, or the palor produced by fright, are illustrations of the influence of the nerves upon the circulation; and there are certain substances that when brought in contact with the epithelium interferes with membranous circulation. Tobacco is a familiar illustration of this fact.

